# *Aquella oligotrophica* gen. nov. sp. nov.: A new member of the family *Neisseriaceae* isolated from laboratory tap water

**DOI:** 10.1002/mbo3.793

**Published:** 2019-01-17

**Authors:** Kok‐Gan Chan, Wah‐Seng See‐Too, Kah‐Ooi Chua, Álvaro Peix, Kian Mau Goh, Kar‐Wai Hong, Wai‐Fong Yin, Li‐Sin Lee

**Affiliations:** ^1^ International Genome Centre Jiangsu University Zhenjiang China; ^2^ Faculty of Science, Institute of Biological Sciences University of Malaya Kuala Lumpur Malaysia; ^3^ Instituto de Recursos Naturales y Agrobiología IRNASA‐CSIC Salamanca Spain; ^4^ Faculty of Sciences Universiti Teknologi Malaysia Skudai Malaysia

**Keywords:** *Aquella oligotrophica* gen. nov. sp. nov., *Neisseriaceae*, OrthoANI, POCP, tap water

## Abstract

A bacterial strain designated as P08^T^ was isolated from laboratory tap water during a water quality assessment in University of Malaya, Malaysia. The strain was a Gram‐negative, rod‐shaped, nonmotile, and aerobic bacterium. Complete genome of P08^T^ comprised of a 2,820,660 bp chromosome with a G + C content of 36.43%. Both 16S rRNA phylogeny and phylogenetic tree inferred from the core gene matrix demonstrated that P08^T^ formed a hitherto unknown subline within the family *Neisseriaceae*. Ortho average nucleotide identity (OrthoANI) values and the percentage of conserved proteins (POCP) calculated from complete genome sequence indicated low relatedness between P08^T^ and its phylogenetic neighbors. Respiratory quinone analysis revealed Q‐8 as the only detectable quinone. The predominant cellular fatty acids were identified as C_14:0_, iso‐C_15:0_, and summed feature 3 (C_16:1_ *ω*7c/C_16:1_ *ω*6c). The polar lipids consisted of uncharacterized aminolipid, phosphatidylglycerol, and phosphatidylethanolamine. All aspects of phenotypic and phylogenetic data suggested that strain P08^T^ represents a novel genus within family *Neisseriaceae*, for which the name *Aquella* gen. nov. is proposed. The type species of the genus is *Aquella oligotrophica *sp. nov., and the type strain is P08^T^ (=LMG 29629^T^ =DSM 100970^T^).

## 1 INTRODUCTION

The order Neisseriales, which constitutes a major branch of *Betaproteobacteria*, presently contains 36 genera spanning a wide range of morphologies, habitats, and growth requirements (Parte, 2013). The taxonomic classification in Neisseriales is primarily based on 16S rRNA sequence identity studies and phylogenetic analysis (Adeolu & Gupta, 2013), which the taxon delineation was mainly determined by distinct clade from the phylogenetic tree. The type family of Neisseriales is *Neisseriaceae *(Tønjum, 2015).

In 2013, Adeolu and Gupta proposed that a new family, *Chromobacteriaceae*, to be split from *Neisseriaceae* based on phylogenomic and molecular signatures evidence (Adeolu & Gupta, 2013). The amendment reclassified 19 genera from 32 genera of *Neisseriaceae* to *Chromobacteriaceae*. The emended *Neisseriaceae *contains *Alysiella *(Langeron, 1923), *Bergeriella* (Xie & Yokota, 2005), *Conchiformibius* (Xie & Yokota, 2005), *Eikenella* (Jackson & Goodman, 1972), *Kingella* (Henriksen & Bøvre, 1976), *Neisseria* (Trevisan, 1885), *Simonsiella* (Simons, 1922), *Stenoxybacter* (Wertz & Breznak, 2007), *Uruburuella* (Vela et al., 2005), and *Vitreoscilla *(Pringsheim, 1949).

Subsequently, 4 additional genera have been validly described and classified into family *Neisseriaceae,* namely *Amantichitinum *(Moß et al., 2013), *Snodgrassella *(Kwong & Moran, 2013), *Rivicola *(Sheu, Chen, Young, & Chen, 2014), *Crenobacter *(Dong et al., 2015), and *Populibacter *(Li, Xue, Sang, Lin, & Wang, 2017). At the time of writing, there are 17 validly described genera included in family *Neisseriaceae. *However, there are also 2 genera with uncertain taxonomic status that have been classified into *Neisseriaceae;* Adeolu and Gupta (2013) suggested that genus *Morococcus* (Long, Sly, Pham, & Davis, 1981) should be reclassified to *Neisseria *species and *Prolinoborus* (Pot, Willems, Gillis, & De Ley, 1992) is likely wrongly assigned to the order of Neisseriales*, *based on their phylogenetic analysis.

Other than 16S rRNA gene sequence similarities and phylogenetic analysis, the major distinguishing features among genera for *Neisseriaceae* are cell morphology, biochemical characteristics, such as oxidase and catalase tests, glucose fermentation, nitrite reduction, and mol% G + C content of the genomic DNA (Garrity et al., 2006). Most strains inhabit indigenously in mucosal membranes of humans and animals, although environmental species were recently included in this family with representatives isolated from anthill, hot spring sediment, and freshwater river (Dong et al., 2015; Garrity et al., 2006; Moß et al., 2013; Sheu et al., 2014). During an assessment on laboratory tap water quality, a bacterial strain designated as P08^T^ was isolated on R2A agar. Here, we report the characterization of strain P08^T^ that is the first representative of a newly proposed genus *Aquella* gen. nov. belonging to family *Neisseriaceae*.

## 2 MATERIALS AND METHODS

### 2.1 Isolation of the strain

Strain P08^T^ was isolated from a laboratory tap water collected for water quality assessment in University of Malaya (3°07′20.9″N, 101°39′23.7″E) on 22 May 2015. After 3 days of incubation on R2A agar (BD Difco) at 37°C, bacteria isolation and purification were performed. The purified strain was routinely cultivated in R2A liquid medium, unless specified. Cells were preserved in 20% (v/v) glycerol at −80°C. The strain P08^T^ has been deposited in the German Collection of Microorganisms and Cell Cultures (DSMZ, Braunschweig, Germany) and the Belgium Coordinated Collections of Microorganisms (BCCM/LMG, Gent, Belgium).

### 2.2 DNA extraction, genome sequencing, and functional gene annotation

The genomic DNA of strain P08^T^ was extracted using the MasterPure^TM^ DNA purification kit (Epicenter, WI, USA) following the manufacturer's protocol. Extracted genomic DNA was sheared and constructed into a template library according to the “Guidelines for Preparing 20 kb SMRTbell™ Templates.” Genome sequencing was performed in 1 SMRT cell using the PacBio RS II single‐molecule real‐time (SMRT) sequencing technology (Pacific Biosciences, CA, USA). The reads were de novo assembled using the hierarchical genome assembly process (HGAP) algorithm version 2 (Chin et al., 2013) into complete genome of P08^T^. The assembled genome was annotated using the NCBI Prokaryotic Genome Annotation Pipeline (PGAP) version 2.10 (Tatusova et al., 2016), Rapid Annotation using Subsystem Technology (RAST) version 3.0 (Aziz et al., 2008; Overbeek et al., 2014), and IMG ER pipeline (Markowitz et al., 2009). The genome project and the complete genome sequence were deposited in the Genomes OnLine Database (Liolios et al., 2010) and GenBank. A comparison on the genomes of P08^T^ and available genomes of type strains in the family *Neisseriaceae* was performed.

### 2.3 Phylogenetic analyses

The 16S rRNA gene sequence was mined from the complete genome using RNAmmer 1.2 server (Lagesen et al., 2007). The sequence similarity to other validly described type strains was examined from pairwise sequence comparisons using EzBioCloud database (https://www.ezbiocloud.net) (Yoon et al., 2017). Phylogenetic analysis of 16S rRNA genes of strain P08^T^ and type members of *Neisseriaceae* were performed using the software package MEGA version 6.0 (Tamura, Stecher, Peterson, Filipski, & Kumar, 2013), with neighbor‐joining (Saitou & Nei, 1987), maximum‐likelihood (Felsenstein, 1981), and maximum parsimony (Fitch, 1971) algorithms. In each case, bootstrap values were calculated based on 1,000 resamplings (Felsenstein, 1985). Kimura's two‐parameter model was used to calculate evolutionary distance matrices of the neighbor‐joining method and maximum‐likelihood method (Kimura, 1980). *Vitreoscilla stercoraria *was included in the analysis in place of the type species *V. beggiatoides* that has no sequence data available. For core genes (present in all genomes) analysis, the homologous clusters were determined using panX pan‐genome pipeline (Ding, Baumdicker, & Neher, 2018). The alignments were then built from these orthologous clusters before being concatenated and used for phylogenetic analysis in RAxML (Stamatakis, 2014). Generally, the phylogenetic tree was constructed by applying GAMMA for modeling rate heterogeneity, fast bootstrapping in conjunction with the autoMRE bootstopping criterion (Pattengale, Alipour, Bininda‐Emonds, Moret, & Stamatakis, 2010), and subsequent search for the best tree (Stamatakis, Hoover, & Rougemont, 2008). The core genes sequences of strain P08^T^ were compared against members of the genera *Alysiella*, *Amantichitinum*, *Bergeriella*, *Conchiformibius*, *Crenobacter*, *Eikenella*, *Kingella*, *Morococcus*, *Neisseria*, *Populibacter, Prolinoborus, Rivicola, Simonsiella*, *Snodgrassella*, *Stenoxybacter,* and *Vitreoscilla*.

### 2.4 Average Nucleotide Identity (ANI), Average Amino acid Identity (AAI), and Percentage of conserved proteins (POCP) analysis

ANI between the complete genome of P08^T^ and each reference genome was calculated using an online ANI calculation tool on the EzBioCloud web server with the OrthoANI algorithm (Lee, Kim, Park, & Chun, 2015). AAI between of complete genome of P08^T^ and each reference genome was calculated using an online AAI calculation tool available at the web server http://enve-omics.ce.gatech.edu/(Rodriguez‐R & Konstantinidis, 2016). The available genome sequences of the family *Neisseriaceae* were retrieved from GenBank. The percentage of conserved proteins (POCP) (Qin et al., 2014) in each pair of genomes was calculated as [(C1+C2)/(T1+T2)]×100%, where C1 and C2 represent the numbers of conserved proteins in the two genomes being compared, respectively, and T1 and T2 represent the total numbers of predicted proteins in the two genomes being compared, respectively. Conserved proteins were defined as having a BLASTP match with an E‐value of less than 1e^−5^, sequence identity of more than 40% and an alignable region of the query protein sequence of more than 50%, as recommended by Qin et al. (2014).

### 2.5 Morphological observations and physiological tests

Growth of strain P08^T^ was tested on chocolate agar (Thermo Scientific), Luria‐Bertani agar (Merck), *Pseudomonas* agar (BD Difco), MacConkey agar (Merck), R2A agar (BD Difco), and trypticase soy agar (Merck), and the ability to grow in this media was recorded after incubation of 3 days at 37 °C. Oxidase and catalase activities were examined with solutions of oxidase reagent (bioMérieux) and 3% (v/v) hydrogen peroxide, respectively. The morphology of bacterial cells was observed using tabletop scanning electron microscopy (Hitachi TM3030, Germany). Cellular motility was tested by the hanging drop method (Beveridge, Lawrence, & Murray, 2007). Gram staining was performed using the standard Gram reaction and was confirmed by using the KOH lysis test method (Cerny, 1978). Growth temperature was investigated in R2A medium at 4, 12, 16, 20, 25, 30, 35, 37, 40, and 45 °C, up to 14 days of incubation. Growth at pH 4.0–11.0 (intervals of 1 unit) was determined in R2A medium after incubation for 14 days at 37 °C under speed 220 rpm. For pH adjustment of the basal medium, the following buffers were used as follows: 0.1 M citric acid/0.1 M sodium citrate (pH 4.0–5.0), 0.1 M KH_2_PO_4_/0.1 M NaOH (pH 6.0–8.0), 0.1 M NaHCO_3_/0.1 M Na_2_CO_3_ (pH 9.0–10.0), and 0.05 M Na_2_HPO_4_/0.1 M NaOH. Growth on medium added with sodium chloride (NaCl) was determined in R2A medium supplemented with 0, 0.5, 1%–6% (intervals of 1%) (w/v) NaCl after 14 days of incubation at 37 °C. Growth under anaerobic condition was determined by incubating strain P08^T^ on R2A agar in the Oxoid AnaeroGen system.

P08^T^ was tested on R2A agar supplemented with casein (2% skimmed milk, w/v), starch (0.2% soluble starch, w/v), cellulose (0.5% CM‐cellulose, w/v), urease, Tweens 20, 40, 60, and 80 for hydrolysis activities, respectively (Tindall, Sikorski, Smibert, & Krieg, 2007). Hydrolysis of DNA was determined using DNase test agar (BD Difco). The production of clear or opaque halo zones around colonies on agar plates was recorded as a positive result after incubation of 3 weeks, except for hydrolysis of starch, cellulose, urease, and DNA (observed on agar plates incubated for 2 weeks). The color and morphology of colonies were determined on R2A agar incubated for 2 days at 37 °C. Other physiological and biochemical tests were performed using API 20E, API 20NE, and API ZYM strips (bioMérieux) according to the manufacturer's instructions, and results were recorded after 2 days of incubation at 37 °C. Utilization of a variety of carbon sources was tested using GEN III MicroPlates (Biolog), along with other major biochemical and physiological properties. Cells grown for 2 days at 37 °C on R2A were suspended in sterilized inoculating fluid C (Biolog) and adjusted to a specific transmittance (60% T) using a turbidimeter according to the manufacturer's instruction. An aliquot (100 μl) of the cell suspension was transferred to each well, and the plate was immediately incubated for 3 days at 37 °C, before visual reading.

### 2.6 Antibiotic susceptibility testing

Sensitivity of strain P08^T^ to antibiotics was tested by the disk diffusion method after spreading cell suspensions (0.5 McFarland) on R2A agar (BD Difco) plates. The disks (Oxoid) contained the following antibiotics: ampicillin (10 μg), ampicillin/sulbactam (20 μg), chloramphenicol (30 μg), gentamicin (10 μg), kanamycin (30 μg), nalidixic acid (30 μg), rifampicin (5 μg), penicillin G (10 μg), streptomycin (10 μg), sulfamethoxazole (23.75 μg) plus trimethoprim (1.25 μg), and tetracycline (30 μg). The effect of antibiotics on cell growth was assessed after incubation for 2 days at 37°C. The diameter of the antibiotic disks was 6 mm. The strain was considered susceptible when the diameter of the inhibition zone was>13 mm, intermediate at 10–12 mm, and resistant at <10 mm as described by Nokhal and Schlegel (1983).

### 2.7 Fatty acid analyses

Fatty acid methyl esters are extracted from 40 mg cells scraped from Petri dishes by saponification, methylation, and extraction using minor modifications of the method of Kuykendall, Roy, O'neill, and Devine (1988) and Miller (1982). Fatty acid methyl esters were separated and analyzed by the Identification Service of the DSMZ, Braunschweig, Germany, using the Sherlock Microbial Identification System (MIDI Inc, Newark, USA, version 6.1 with database TSBA6) according to the standard protocol. For this purpose, strain P08^T^ was grown for 2 days on R2A plates at 37 °C to get cultures of the same physiological age.

### 2.8 Polar lipid and respiratory quinones analyses

Polar lipid analyses and analyses of respiratory quinones were carried by the Identification Service of the DSMZ, Braunschweig, Germany. Cells were freeze‐dried before 100 mg cell material was used for extraction of respiratory lipoquinones using the two‐stage method described by Tindall (1990a) and Tindall (1990b). Respiratory quinones were then extracted using methanol:hexane, followed by phase separation into hexane (Tindall, 1990a, 1990b). Separation of respiratory lipoquinones into different classes was conducted by thin layer chromatography (TLC) on silica gel (Macherey‐Nagel Art. No. 805 023) using hexane:tertbutylmethylether [9:1 (v/v)] as the solvent. UV‐absorbing bands corresponding to different quinone classes were removed from the TLC plate and further analyzed on a LDC Analytical (Thermo Separation Products) HPLC fitted with a reverse phase column (Macherey–Nagel, 2 mm × 125 mm, 3 μm, RP18) using methanol:heptane [9:1 (v/v)] as the eluent. Respiratory lipoquinones were detected at 269 nm. Cells were freeze‐dried before 100 mg cell material was used for polar lipids extraction with a modified method using a chloroform:methanol:0.3% aqueous NaCl mixture 1:2:0.8 (v/v/v) (Bligh & Dyer, 1959). The mixture was stirred overnight, and cell debris was centrifuged for pellet. Polar lipids were obtained from the chloroform phase by adjusting the chloroform:methanol:0.3% aqueous NaCl mixture to a ratio of 1:1:0.9 (v/v/v). Recovered polar lipids were separated by a two‐dimensional silica gel TLC (Macherey‐Nagel Art. No. 818 135), first in chloroform:methanol:water (65:25:4, v/v/v) and the second in chloroform:methanol:acetic acid:water (80:12:15:4, v/v/v/v). Total lipid material was detected using molybdatophosphoric acid while specific functional groups detected using spray reagents specific for defined functional groups (Tindall et al., 2007).

## 3 RESULTS

### 3.1 Phylogenetic analyses

**Figure 1:**
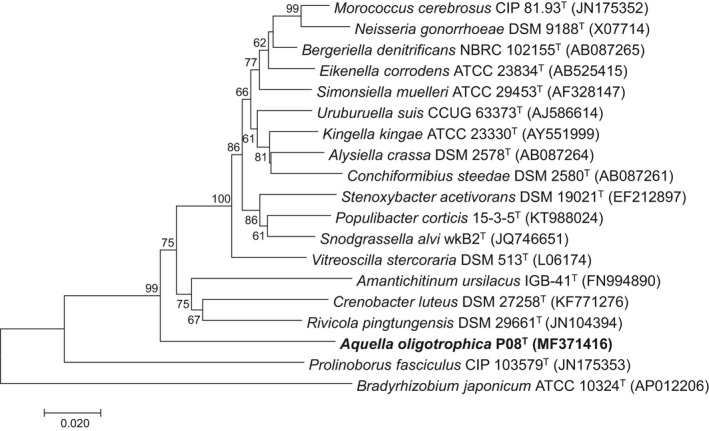
16s rRNA gene phylogeny of strain P08^T^ and other members of *Neisseriaceae*. Neighbor‐joining phylogenetic tree based on 16S rRNA gene sequences showing the positions of strain P08^T^ (MF371416) and the most closely related members of the family *Neisseriaceae*. Bootstrap percentage values (1,000 replications) greater than or equal to 50% are shown at nodes. *Bradyrhizobium japonicum* ATCC 10324^T^ (U69638) is used as an out‐group. Bar, 0.02 substitutions per nucleotide position

**Figure 2:**
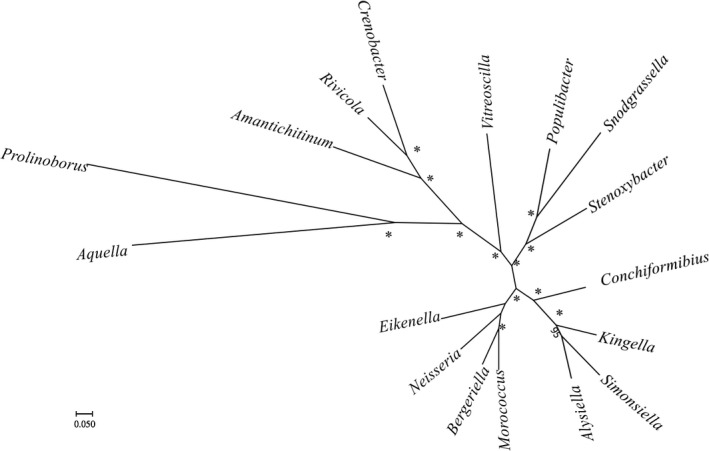
Phylogeny of the *Neisseriaceae*. ML tree reconstructed from concatenated core gene sequences (146 genes, 133,677 characters). Only bootstrap values of 50% or greater are shown. Asterisks adjacent to nodes indicated 100% bootstrap support. Bar, 0.05 substitutions per nucleotide position

Complete 16S rRNA gene sequence of strain P08^T^ is 1,532 bp. The 16S rRNA gene sequence similarity was highest to *Neisseria animaloris* LMG 23011^T^ (90.1%), and lesser to all validly described type strains, for instance *Neisseria iguana* NVSL 85737^T^ (89.4%), *Neisseria flavescens* ATCC 13120^T^ (89.3%), *Paludibacterium paludis* KBP‐21^T^ (89.3%), *Uruburuella testudines* 07_OD624^T^ (89.3%), and *Morococcus cerebrosus* CIP 81.93^T^ (89.3%) based on EzBioCloud similarity‐based search (Table S1). According to Ludwig et al. (1998), 16S rRNA gene sequence similarities of lesser than 95% between two bacteria are generally affiliated as a novel genus. Therefore, the low 16S rRNA gene sequence similarity to other validly described type strains highly supported that strain P08^T^ belongs to a novel genus. Figure 1 shows the positions of the type species of genera within the family *Neisseriaceae* in a neighbor‐joining (NJ) phylogenetic tree based on 16S rRNA gene sequences. Strain P08^T^ formed a branch clearly separated from the remaining genera from *Neisseriaceae*. As indicated in Figure 1, strain P08^T^ appeared to be more closely related to *Neisseriaceae*, compared to the type species of genus *Prolinoborus, *which have a questionable taxonomic status in *Neisseriaceae* (Adeolu & Gupta, 2013). The NJ phylogenetic tree was congruent with the maximum‐likelihood (ML) (Figure S1) and maximum parsimony (MP) (Figure S2) phylogenetic trees, all constructed based on 16S rRNA gene sequences. For core genes analysis, we have identified 146 orthologous protein clusters (Table S2). The ML phylogenetic tree based on concatenated core gene (Figure 2) showed that all genera in the *Neisseriaceae* formed distinct phylogenetic lineages. Strain P08^T^ was in a cluster distantly related to *Snodgrassella *and was clearly separated in the phylogenetic tree based on concatenated core gene from other genera of *Neisseriaceae*. This association was supported by a bootstrap value of 100%. Likewise, the concatenated core gene phylogenetic tree was congruent with the 16S rRNA gene phylogenetic analyses in which strain P08^T^ represents a novel bacterial taxon in the family *Neisseriaceae.*

### 3.2 Genomic features and functional gene annotation

**Figure 3:**
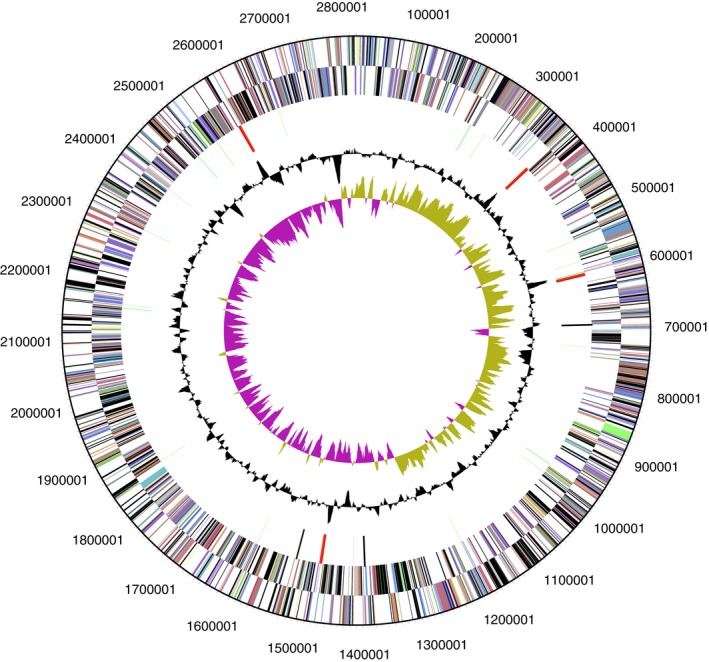
Circular map of the chromosome and plasmid of *A. oligotrophica* P08^T^. From the outside to the centre: genes on forward strand (colored by COG categories), genes on reverse strand (colored by COG categories), RNA genes (tRNAs green, rRNAs red, other RNAs black), GC content, and GC skew

A total of 1,985,842,173 reads with a mean read length of 11,876 bp were generated from whole genome sequencing. The reads were de novo assembled using the hierarchical genome assembly process (HGAP) algorithm version 2 (Chin et al., 2013) to generate the complete genome of P08^T^, which consists of 1 polished contig with an average genome coverage of 472.61‐fold. The genome of P08^T^ is 2,820,660 bp in length, consists of a circular chromosome, with G + C contents of 36.43 mol%. The assembled and annotated genome of P08^T^ described in this paper has been deposited in GenBank (accession number: CP024847.1) and JGI portal (GOLD ID: Gp0293937; IMG Taxon ID: 2,770,939,448). Out of a total of 2,625 genes in the genome, 2,564 protein‐coding gene, 12 rRNAs, and 46 tRNAs were predicted from the chromosome by PGAP analysis (Table 1 and Figure 3). Complete genome sequencing analysis revealed that the protein‐coding gene constituted 97.68% of the total genes in the genome of P08^T^ but only 76.11% are predicted with functions. Notably, several genes that confer resistance to beta‐lactam antibiotics were also identified in the genome and were annotated as class A beta‐lactamase [NCBI locus tag =CUN60_09190] and class D beta‐lactamase [CUN60_03820]. Furthermore, there were 1621 genes assigned to different functional categories based on the clusters of orthologous genes (COG) designation (Tatusov, Galperin, Natale, & Koonin, 2000), 756 genes were connected to KEGG pathways, and 619 genes connected to MetaCyc pathways (Table 1). Majority of the genes were categorized into classes responsible for central metabolism of the P08^T ^including translation, ribosomal structure and biogenesis and amino acid transport and metabolism. There are also a number of genes with unknown functions. Figure S3 represents the gene's distribution into different clusters of orthologous groups (COGs) functional categories.

**Table 1:** Genome statistics of *Aquella oligotrophica* gen. nov. sp. nov. (strain P08^T^)

Attribute	Value	% of total
Genome size (bp)	2,820,660	100.00
DNA coding region (bp)	2,571,724	91.17
DNA G + C content (bp)	1,027,475	36.43
Total genes	2,625	100.00
Protein‐coding gene	2,564	97.68
RNA genes	61	2.32
rRNA genes	12	0.46
tRNA genes	46	1.75
Genes with function prediction	1998	76.11
Genes assigned to COGs	1621	61.75
Genes assigned to KEGG pathways	756	28.80
Genes assigned to MetaCyc pathways	619	23.58

### 3.3 Genome comparisons with type strains of type species in the family *Neisseriaceae*

Genome of P08^T^ was compared to genome of other members in the family *Neisseriaceae*. With the exception to *Crenobacter luteus*, *Vitreoscilla beggiatoides, *and *Uruburuella suis*, publicly available genomes of all type strains of type species in the family were included. Genome of *C. luteus* strain CN10 was selected as the type strain of the type species *C. luteus* has no available genome information while the genome of *V. stercoraria* DSM 513^T^ was incorporated into analysis as the type species *V. beggiatoides* has no available culture. *Uruburuella *is the only genus currently without genomic data. Most of the genomes currently available were draft genomes, and complete genomes obtained were only limited to strains P08^T^, *Simonsiella muelleri* ATCC 29453^T^, and *Snodgrassella alvi* wkB2^T ^(Table S3). While an accurate comparison on the size would require the complete genomes of the other type strains, it is noteworthy that the complete genome size of P08^T^ is 2.82 Mb, which is among the largest and only smaller than the genome size of *Amantichitinum ursilacus* IGB‐41^T^, *Crenobacter luteus* CN10, *Prolinoborus fasciculus *CIP103579^T^, and *Rivicola pingtungensis *DSM 29661^T^ (4.93, 2.85, 3.45, and 3.71 Mb, respectively). Intriguingly, strain P08^T^ has the lowest G + C content among these genomes. The range of G + C contents ranged widely from the lowest 36.43% in strain P08^T^ to the highest 68.30% in genome of *Crenobacter luteus* CN10 (Table S3).

### 3.4 Average Nucleotide Identity (ANI), Average Amino acid Identity (AAI), and Percentage of Conserved Proteins (POCP) analysis

Genome‐to‐genome similarity between strain P08^T^ and close taxa was performed using OrthoANI algorithm (Lee et al., 2015). OrthoANI values between strain P08^T^ and phylogenetically related genera in *Neisseriaceae *ranged from 63.0% to 65.5% (Table 2), which were significantly below the proposed boundary of 95%–96% for defining a novel species (Richter & Rosselló‐Móra, 2009). Despite ANI is not suitable for genus delimitation (Qin et al., 2014), this analysis briefly suggested that strain P08^T^ is not the species or the subspecies of aforementioned taxa. Similar observation was obtained from AAI analysis, and strain P08^T^ has AAI values ranged from 40.62% to 46.71% with all reference genomes, which is much lower than 95%–96% for proposing a novel species (Table S4) (Konstantinidis & Tiedje, 2005). POCP has been applied to support the designation of P08^T ^as a novel genus, using a suggested threshold value of 50% (Qin et al., 2014). POCP using the cutoff threshold for genus circumscription highly supported that strain P08^T^ represented a novel genus in family *Neisseriaceae*, as strain P08^T^ had an extremely low POCP compared to other representative members of family *Neisseriaceae.* The intergenera POCP analyses between P08^T^ and strains from 13 genera of family *Neisseriaceae *had values ranged from 24% to 31%, which clearly indicated that strain P08^T^ does not belong to any validly described genera from *Neisseriaceae* (Table 2).

**Table 2:** OrthoANI and POCP values for pairs of genomes between strain P08^T^ and other phylogenetically related genera in the family *Neisseriaceae*

Bacteria	OrthoANI (%)	POCP (%)
*Alysiella crassa* DSM 2578^T^	64.0	23
*Amantichitinum ursilacus* IGB−41^T^	63.4	23
*Bergeriella denitrificans* NBRC 102155^T^	63.7	27
*Conchiformibius steedae* DSM 2580^T^	64.4	26
*Crenobacter luteus* CN10	63.0	29
*Eikenella corrodens* ATCC 23834^T^	63.9	26
*Kingella kingae* ATCC 23330^T^	64.5	27
*Morococcus cerebrosus* CIP 81.93^T^	64.4	27
*Neisseria gonorrhoeae* DSM 9188^T^	64.1	26
*Populibacter corticis *15–3−5^T^	64.6	25
*Prolinoborus fasciculus *CIP 103579^T^	63.6	19
*Rivicola pingtungensis *DSM 29661^T^	63.1	26
*Simonsiella muelleri* ATCC 29453^T^	64.7	25
*Snodgrassella alvi* wkB2^T^	65.5	26
*Stenoxybacter acetivorans *DSM 19021^T^	64.6	25
*Vitreoscilla stercoraria *DSM 513^T^	64.5	26

### 3.5 Fatty acid, polar lipid, and respiratory quinones composition

**Figure 4:**
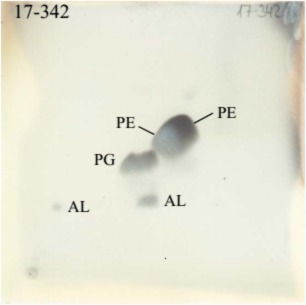
Thin layer chromatography image of polar lipid analysis of P08^T^; AL, aminolipid; PG, phosphatidylglycerol; PE, phosphatidylethanolamine

The fatty acid composition of strain P08^T^ is listed in Table 3. The major fatty acids (>5%) were C_14:0_ (15.9%), iso‐C_15:0_ (36.2%), C_16:0_ (9.9%), summed feature 3 (C_16:1_ *ω*7c/C_16:1_ *ω*6c) (16.4%), and summed feature 8 (C_18:1_ *ω*7c) (6.8%). Strain P08^T^ exhibited a polar lipid profile consisting of uncharacterized aminolipid, phosphatidylglycerol, and phosphatidylethanolamine (Figure 4). Strain P08^T^ had Q‐8 as the major respiratory quinone, which is the same as its closest phylogenetic relatives (Dong et al., 2015; Moß et al., 2013; Sheu et al., 2014; Srinivas et al., 2013; Su et al., 2013).

**Table 3:** Cellular fatty acid compositions of *Aquella oligotrophica* gen. nov. sp. nov. strain P08^T^

Fatty acid	Composition
Straight‐chain	
C_12:0_	–
C_14:0_	15.9
C_16:0_	9.9
Branched‐chain	
iso‐C_15:0_	36.2
C_17:0_ cyclo	–
Unsaturated	
C_14:1_ *ω*5c	4.7
C_15:1_ *ω*6c	1.7
C_16:1_ *ω*7c	–
C_18:1_ *ω*7c	–
Hydroxy	
C_12:0_ 3‐OH	–
C_16:0_ 3‐OH	1.4
Summed features^a^	
3	16.4
8	6.8
9	1.9

Note

Strain P08^T ^was grown on R2A agar at 37 °C for 2 days. Values are in percentages of the total fatty acids; fatty acids that make up <1% of the total are not listed or are indicated by a dash.

a Summed features represent groups of two or three fatty acids that cannot be separated by GLC with the MIDI system. Summed feature 3 comprises C_16:1_ *ω*7c and/or C_16:1_ *ω*6c; summed feature 8 comprises C_18:1_ *ω*7c and/or C_18:1_ *ω*6c.

## 4 DISCUSSION

The 16S rRNA gene sequence similarity of strain P08^T^ with other validly described strains is lower than the proposed cutoff (95%) value suggested by Ludwig et al. (1998) for a novel bacterial genus. EzBioCloud similarity‐based search in Table S1 shows that strain P08^T^ has highest 16S rRNA gene sequence similarity with most of the type strains of species belonging to the genus *Neisseria*. This suggested that even though strain P08^T^ does not belong to any validly described genera, it is highly related to family *Neisseriaceae*. The divergent branching pattern between strain P08^T^ and the cluster of genera of the family *Neisseriaceae* was highly reproducible with solid bootstrap recovery using the neighbor‐joining algorithm (Figure 1). This was further confirmed in phylogenetic tree constructed using concatenated core genes (Figure 2). In the phylogenetic tree based on concatenated core genes, 146 orthologous protein clusters were concatenated and subjected to ML analysis. Strain P08^T^ is distantly related to other members of family *Neisseriaceae*. Interestingly, even though Adeolu and Gupta (2013) proposed that *Moroccocus *should be reclassified into genus *Neisseria*, phylogenetic tree based on concatenated core genes showed that *Moroccocus *is rather closely related to *Bergeriella*. However, these 3 taxa clustered into the same clade and showed that they are more related to each other than to other members from family *Neisseriaceae.*

We reported in this study the complete genome sequence of P08^T^ that is important for pairwise comparison with closely related species in terms of ANI and AAI analyses. The level of genome‐to‐genome similarity between strain P08^T^ and the closest phylogenetic neighbors was significantly below the interspecies level of sequence similarity (Table 2). The genus delimitation as determined by POCP between strain P08^T^ and *Neisseria gonorrhoeae *DSM 9188^T^, *Amantichitinum ursilacus* IGB‐41^T^, and *Crenobacter luteus* CN10 was of values 26%, 23%, and 29%, respectively (Table 2). This is far below the proposed threshold for genus delimitation of 50% (Qin et al., 2014), which indicates that strain P08^T^ does not belong to any of these mentioned genera. In fact, the relatedness among all these genome sequences is extremely low, which also suggests that strain P08^T^ represents a novel genus.

Several beta‐lactamase gene were identified in the genome of P08^T^, and the drug‐resistant phenotype of P08^T^ toward antibiotics was furthered confirmed by antibiotic susceptibility test. Consistent with the complete genome analysis, P08^T^ was resistant to beta‐lactam antibiotics including ampicillin and penicillin G tested in this study. The class A and D beta‐lactamases identified are known to confer broad‐spectrum resistance toward beta‐lactam antibiotics (Bush & Jacoby, 2010).

The predominant fatty acids of strain P08^T^ consisted of C_14:0_, iso‐C_15:0_, and summed feature 3 (C_16:1_ *ω*7c/C_16:1_ *ω*6c) (Table 3). Polar lipid analyses using two‐dimensional TLC (Figure 4) revealed differences between the polar lipid composition of cell membrane of strains P08^T^ and phylogenetically related genera. Strain P08^T^ harbored uncharacterized aminolipid, phosphatidylglycerol, and phosphatidylethanolamine. No unidentified phospholipid was found in P08^T^.

Physiological and biochemical characteristics such as absence of catalase and oxidase, rod‐shaped, absence of motility, inability to reduce nitrate to nitrite, low DNA G + C content, and cellular fatty acid profile clearly distinguished strain P08^T^ from other members of the family *Neisseriaceae* (Table 3 & 4). The ability to grow in up to 43°C is also useful in differentiating strain P08^T^ from the genera *Amantichitinum* and *Rivicola* (Table 4). Based on these morphological, physiological, chemotaxonomic, phylogenetic, and phylogenomic properties, strain P08^T^ is considered to represent a novel species within a new genus, for which the name *Aquella oligotrophica* gen. nov., sp. nov. is proposed.

**Table 4:** Characteristics that differentiate *Aquella oligotrophica* P08^T^ from other phylogenetically related genera in the family *Neisseriaceae*

Characteristics	1	2	3	4	5
Isolation source	Tap water	Thumb wound	Ant hill soil	Hot spring sediment	Freshwater river
Colony pigmentation	Milky white	Opaque, shiny	Milky white to beige	Yellowish	Cream
Cell size (μm)	0.7–1.4 × 0.3–0.5	NA	0.7–0.8 × 1.5–3.0	ND	0.3–0.6 × 1.4–3.2
Cell shape	Rod‐shaped	Circular	Rod‐shaped	Short‐rod‐shaped	Rod‐shaped
Motility	‒	‒	+	+	‒
Oxygen requirement	Strictly aerobic	Facultative anaerobic	Facultative anaerobic	Strictly aerobic	Facultative anaerobic
Temperature range for growth (°C) (optimal)	13‒43 (35‒40)	37 (18‒22)	10‒35 (20‒25)	10‒55 (40‒50)	10‒37 (30‒35)
pH range for growth (optimal)	5.0‒8.0 (5.0‒7.0)	ND	6.0‒9.0 (7.0)	6.0‒10.0 (8.0‒9.0)	6.0‒8.0 (6.0‒7.0)
Salinity range for growth (w/v,%)	0‒1 (0‒1)	ND	0	0‒3 (0‒1)	0‒1 (0)
Nitrate reduction	‒	+	+	+	+
DNA G + C content (mol%)	36.4	49.3	61.5	67.3	64.1
Production of					
Catalase	‒	+	+	+	+
Oxidase	‒	+	+	+	+
Hydrolysis of starch	‒	‒	+	‒	‒
Fermentation of					
Arabinose	+	‒	+	‒	+
D‐mannitol	+	‒	+	‒	+
Maltose	+	‒	+	+	+
Polar lipids	Uncharacterized aminolipid, phosphatidylglycerol, phosphatidylethanolamine	ND	Phosphoaminolipids, aminolipids, glycoaminolipids	Diphosphatidylglycerol, phosphatidylethanolamine, phosphatidylmethylethanolamine, phospholipids of unknown structure containing aminoglycophospholipid, three unidentified polar lipids.	Phosphatidylethanolamine,phosphatidylglycerol, diphosphatidylglycerol, an uncharacterized aminolipid, three uncharacterized phospholipids
Major quinones	Q−8	ND	Q−8	Q−8	Q−8

Note

Taxa: 1, *Aquella oligotrophica* gen. nov. sp. nov. (strain P08^T^); 2, *Neisseria* (Vandamme, Holmes, Bercovier, & Coenye, 2006); 3, *Amantichitinum* (Moß et al., 2013); 4, *Crenobacter* (Dong et al., 2015); 5, *Rivicola* (Sheu et al., 2014). +, Positive reaction; ‒, negative reaction; Q, quinone; ND, not determined.

### 4.1 Description of *Aquella* gen. nov

*Aquella* gen. nov. (A.qu.el'la. L. fem. dim. n. aquella, water, referring to the source of isolation of the novel organism).

Cells are Gram‐negative, aerobic, nonmotile, rod‐shaped. Cells are oxidase and catalase negative. Q‐8 is the only quinone type. Major fatty acids (>10%) are C_14:0_, iso‐C_15:0_, and summed feature 3 (C_16:1_ *ω*7c/C_16:1_ *ω*6c). The main polar lipids consist of uncharacterized aminolipid, phosphatidylglycerol, and phosphatidylethanolamine. The G + C content of the DNA of the type strain of the type species is 36.43 mol%. Based on 16S rRNA sequence analyses, P08^T^ belongs to the *Betaproteobacteria*. The type species is *Aquella oligotrophica* P08^T^.

### 4.2 Description of *Aquella oligotrophica *sp. nov

*Aquella oligotrophica *(ol.i.go.tro.phi'ca. Gr. adj. *oligos*, few; Gr. adj. *trophikos*, nursing, tending or feeding; N.L. fem. adj. *oligotrophica*, eating little, referring to the bacterium not growing in rich media).

**Figure 5:**
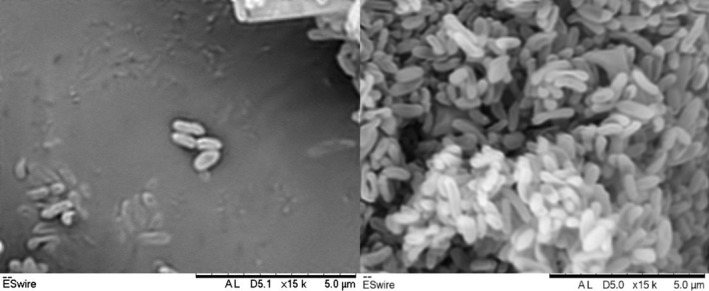
Scanning electron micrographs of strain P08^T^

Exhibits the following properties in addition to those given in the genus description. Cell sizes range from 0.7–1.4 μm in length and 0.3–0.5 μm in width (Figure 5). Cells occur singly, in pairs, in short chains or in irregular clusters. On R2A agar, colonies (<1 mm in diameter) are milky white, circular, and convex with entire margin. Growth occurs at 12–43 °C (optimal growth at 35–40 °C) and at pH 5.0–8.0 (optimal growth at pH 5.0–7.0). Growth occurs in the presence of NaCl up to 1% (w/v). Negative for oxidase and catalase tests. Positive for hydrolysis of casein and Tween 20, 40, 60, and 80, but negative for hydrolysis of starch, CM‐cellulose, urea, and DNA. Based on testing with API 20NE and API 20E, positive reactions towards 4‐nitrophenyl‐β‐_D_‐galactopyranoside, 2‐nitrophenyl‐β‐_D_‐galactopyranoside, and sodium pyruvate. Negative reactions toward potassium nitrate, _L_‐tryptophane, _D_‐glucose, _L_‐arginine, urea, esculin, ferric citrate, gelatin (bovine origin), _D_‐glucose, _L_‐arabinose, _D_‐mannose, _D_‐mannitol, N‐acetyl‐glucosamine, _D_‐maltose, potassium gluconate, capric acid, malic acid, trisodium citrate, phenylacetic acid, _L_‐lysine, _L_‐ornithine, sodium thiosulfate, inositol, _D_‐sorbitol, _L_‐rhamnose, _D_‐sucrose, _D_‐melibiose, and amygdalin. Based on testing with API ZYM kit, C4 esterase, C8 esterase lipase, leucine arylamidase, acid phosphatase, naphthol‐AS‐BI‐phosphohydrolase, *β*‐galactosidase, and N‐acetyl‐β‐glucosaminidase activities are detected but negative for activities of C14 lipase, valine arylamidase, trypsin, α‐chymotrypsin, β‐glucuronidase, α‐glucosidase, β‐glucosidase, α‐mannosidase, and α‐fucosidase. Weak enzymatic activities of alkaline phosphatase, cystine arylamidase, and α‐galactosidase are detected. The major fatty acids (> 5%) are C_14:0_, iso‐C_15:0_, C_16:0_, summed feature 3 (C_16:1_ *ω*7c/C_16:1_ *ω*6c), and summed feature 8 (C_18:1_ *ω*7c). The following compounds are utilized as sole carbon sources in the GEN III microplate: _D_‐galactose, _D_‐glucuronic acid, _D_‐mannose, glucuronamide, glycyl‐_L_‐proline, inosine, _L_‐alanine, _L_‐arginine, _L_‐aspartic acid, _L_‐glutamic acid, _L_‐serine, methyl pyruvate, N‐acetyl‐_D_‐glucosamine, α‐_D_‐glucose, and β‐hydroxy‐_D_,_L_‐butyric acid. All other substrates in the GEN III microplate are not utilized. Sensitive to ampicillin/sulbactam (20 μg), chloramphenicol (30 μg), nalidixic acid (30 μg), rifampicin (5 μg), sulfamethoxazole (23.75 μg) plus trimethoprim (1.25 μg), and tetracycline (30 μg).

The type strain *Aquella oligotrophica* P08^T^ (=LMG 29629^T^ =DSM100970^T^) was isolated from laboratory tap water collected at University of Malaya, Malaysia.

## CONFLICT OF INTERESTS

The authors declare no conflict of interest.

## AUTHORS CONTRIBUTION

KGC supervised the project. LSL carried out the experiments. WSST and LSL wrote the manuscript with support from KGC, KOC, AP, KMG, KWH, and WFY. WSST and LSL analyzed the data.

## ETHICS STATEMENT

None required.

## Data Availability

The assembled and annotated genome of P08T described in this paper has been deposited in GenBank (accession number: CP024847.1) and JGI portal (GOLD ID: Gp0293937; IMG Taxon ID: 2,770,939,448).
