# Supplementary material for: Aquella oligotrophica gen. nov. sp. nov.: A new member of the family Neisseriaceae isolated from laboratory tap water
Source: Microbiologyopen. 2019 Jan 17;8(7):e00793. doi: 10.1002/mbo3.793 (PMC6612550; doi:10.1002/mbo3.793)
Supplement: Supplementary file 1 [file MBO3-8-e00793-s001.docx]

***Aquella oligotrophica* gen. nov. sp. nov., a new member of the family *Neisseriaceae* isolated from laboratory tap water**

**Kok-Gan Chan**^1,2^**, Wah Seng See-Too**^2^**, Kah-Ooi Chua**^2^**, Álvaro Peix**^3^**, Kian Mau Goh**^4^**, Kar-Wai Hong**^2^**, Wai-Fong Yin**^2^ **and Li-Sin Lee**^2^

| ^1^ | International Genome Centre, Jiangsu University, Zhenjiang 212013, People’s Republic of China. |
| --- | --- |
| ^2^ | Institute of Biological Sciences, Faculty of Science, University of Malaya, 50603 Kuala Lumpur, Malaysia. |
| ^3^ | Instituto de Recursos Naturales y Agrobiología, IRNASA-CSIC, Salamanca, Spain. |
| ^4^ | Faculty of Biosciences and Medical Engineering, Universiti Teknologi Malaysia, 81310 Skudai, Johor, Malaysia. |
|  |  |
|  | Correspondence  Kok-Gan Chan  Institute of Biological Sciences, Faculty of Science, University of Malaya, 50603 Kuala Lumpur, Malaysia.  Tel: +603-79677748; Fax:+603-7967727  Email: kokgan@um.edu.my |
|  |  |

| **Rank** | **Name** | **Strain** | **Accession** | **Pairwise Similarity (%)** | **Taxonomy** |
| --- | --- | --- | --- | --- | --- |
| 1 | *Neisseria animaloris* | LMG 23011 | DQ006842 | 90.14 | Bacteria; Proteobacteria; Betaproteobacteria;Neisseriales; *Neisseriaceae*; *Neisseria* |
| 2 | *Neisseria iguanae* | NVSL 85737 | GU233442 | 89.41 | Bacteria; Proteobacteria; Betaproteobacteria; Neisseriales; *Neisseriaceae*; *Neisseria* |
| 3 | *Neisseria flavescens* | ATCC 13120 | L06168 | 89.34 | Bacteria; Proteobacteria; Betaproteobacteria; Neisseriales; *Neisseriaceae*; *Neisseria* |
| 4 | *Paludibacterium paludis* | KBP-21 | HE981224 | 89.34 | Bacteria; Proteobacteria; Betaproteobacteria; Neisseriales; *Neisseriaceae*; *Paludibacterium* |
| 5 | *Uruburuella testudinis* | 07_OD624 | JX966318 | 89.30 | Bacteria; Proteobacteria; Betaproteobacteria; Neisseriales; *Neisseriaceae*; *Uruburuella* |
| 6 | *Morococcus cerebrosus* | CIP 81.93 | JUFZ01000072 | 89.28 | Bacteria; Proteobacteria; Betaproteobacteria; Neisseriales; *Neisseriaceae*; *Neisseria* |
| 7 | *Neisseria macacae* | ATCC 33926 | AFQE01000146 | 89.28 | Bacteria; Proteobacteria; Betaproteobacteria; Neisseriales; *Neisseriaceae*; *Neisseria* |
| 8 | *Neisseria elongata* subsp. *elongata* | ATCC 25295 | L06171 | 89.25 | Bacteria; Proteobacteria; Betaproteobacteria; Neisseriales; *Neisseriaceae*; *Neisseria;Neisseria elongata* |
| 9 | *Neisseria sicca* | ATCC 29256 | ACKO02000016 | 89.21 | Bacteria; Proteobacteria; Betaproteobacteria; Neisseriales; *Neisseriaceae*; *Neisseria* |
| 10 | *Kingella oralis* | ATCC 51147 | ACJW02000005 | 89.21 | Bacteria; Proteobacteria; Betaproteobacteria; Neisseriales; *Neisseriaceae*; *Kingella* |
| 11 | *Neisseria weaveri* | LMG 5135 | AFWQ01000032 | 89.07 | Bacteria; Proteobacteria; Betaproteobacteria; Neisseriales; *Neisseriaceae*; *Neisseria* |
| 12 | *Neisseria elongata* subsp*. glycolytica* | ATCC 29315 | ADBF01000003 | 89.07 | Bacteria; Proteobacteria; Betaproteobacteria; Neisseriales; *Neisseriaceae*; *Neisseria;Neisseria elongata* |
| 13 | *Neisseria canis* | ATCC 14687 | L06170 | 89.04 | Bacteria; Proteobacteria; Betaproteobacteria; Neisseriales; *Neisseriaceae*; *Neisseria* |
| 14 | *Alysiella filiformis* | ATCC 15532 | AF487710 | 89.00 | Bacteria; Proteobacteria; Betaproteobacteria; Neisseriales; *Neisseriaceae*; *Alysiella* |
| 15 | *Alysiella crassa* | DSM 2578 | JQKH01000114 | 89.00 | Bacteria; Proteobacteria; Betaproteobacteria; Neisseriales; *Neisseriaceae*; *Alysiella* |
| 16 | *Neisseria cinerea* | ATCC 14685 | ACDY02000019 | 89.00 | Bacteria; Proteobacteria; Betaproteobacteria; Neisseriales; *Neisseriaceae*; *Neisseria* |
| 17 | *Kingella denitrificans* | ATCC 33394 | GL870930 | 89.00 | Bacteria; Proteobacteria; Betaproteobacteria; Neisseriales; *Neisseriaceae*; *Kingella* |
| 18 | *Neisseria shayeganii* | 871 | AGAY01000088 | 89.00 | Bacteria; Proteobacteria; Betaproteobacteria; Neisseriales; *Neisseriaceae*; *Neisseria* |
| 19 | *Neisseria zoodegmatis* | LMG 23012 | DQ006843 | 88.97 | Bacteria; Proteobacteria; Betaproteobacteria; Neisseriales; *Neisseriaceae*; *Neisseria* |
| 20 | *Kingella kingae* | ATCC 23330 | GL891966 | 88.93 | Bacteria; Proteobacteria; Betaproteobacteria; Neisseriales; *Neisseriaceae*; *Kingella* |
| 21 | *Bergeriella denitrificans* | NBRC 102155 | BCWL01000265 | 88.93 | Bacteria; Proteobacteria; Betaproteobacteria; Neisseriales; *Neisseriaceae*; *Bergeriella* |
| 22 | *Neisseria oralis* | 6332 | JN104029 | 88.93 | Bacteria; Proteobacteria; Betaproteobacteria; Neisseriales; *Neisseriaceae*; *Neisseria* |
| 23 | *Neisseria wadsworthii* | 9715 | AGAZ01000083 | 88.80 | Bacteria; Proteobacteria; Betaproteobacteria; Neisseriales; *Neisseriaceae*; *Neisseria* |
| 24 | *Rivicola pingtungensis* | Npb-03 | JN104394 | 88.74 | Bacteria; Proteobacteria; Betaproteobacteria; Neisseriales; *Neisseriaceae*; *Rivicola* |
| 25 | *Leeia oryzae* | DSM 17879 | AQXU01000006 | 88.73 | Bacteria; Proteobacteria; Betaproteobacteria; Neisseriales; *Neisseriaceae*; *Leeia* |
| 26 | *Neisseria polysaccharea* | ATCC 43768 | ADBE01000137 | 88.73 | Bacteria; Proteobacteria; Betaproteobacteria; Neisseriales; *Neisseriaceae*; *Neisseria* |
| 27 | *Aquaspirillum serpens* | IAM 13944 | AB074518 | 88.66 | Bacteria; Proteobacteria; Betaproteobacteria; Neisseriales; *Neisseriaceae*; *Aquaspirillum* |
| 28 | *Oxalobacter vibrioformis* | DSM 5502 | FR733700 | 88.66 | Bacteria; Proteobacteria; Betaproteobacteria; Burkholderiales; Oxalobacteraceae; *Oxalobacter* |
| 29 | *Eikenella corrodens* | ATCC 23834 | ACEA01000028 | 88.59 | Bacteria; Proteobacteria; Betaproteobacteria; Neisseriales; *Neisseriaceae*; *Eikenella* |
| 30 | *Neisseria bacilliformis* | ATCC BAA-1200 | GL878495 | 88.59 | Bacteria; Proteobacteria; Betaproteobacteria; Neisseriales; *Neisseriaceae*; *Neisseria* |
| 31 | *Neisseria meningitidis* | MC58 | AE002098 | 88.52 | Bacteria; Proteobacteria; Betaproteobacteria; Neisseriales; *Neisseriaceae*; *Neisseria* |
| 32 | *Gulbenkiania mobilis* | E4FC31 | AM295491 | 88.49 | Bacteria; Proteobacteria; Betaproteobacteria; Neisseriales; *Neisseriaceae*; *Gulbenkiania* |
| 33 | *Vitreoscilla stercoraria* | DSM 513 | ARNN01000006 | 88.45 | Bacteria; Proteobacteria; Betaproteobacteria; Neisseriales; *Neisseriaceae*; *Vitreoscilla* |
| 34 | *Snodgrassella alvi* | wkB2 | CP007446 | 88.38 | Bacteria; Proteobacteria; Betaproteobacteria; Neisseriales; *Neisseriaceae*; *Snodgrassella* |
| 35 | *Neisseria lactamica* | ATCC 23970 | ACEQ01000095 | 88.38 | Bacteria; Proteobacteria; Betaproteobacteria; Neisseriales; *Neisseriaceae*; *Neisseria* |
| 36 | *Neisseria elongata* subsp. *nitroreducens* | CIP 103511 | JN175349 | 88.26 | Bacteria; Proteobacteria; Betaproteobacteria; Neisseriales; *Neisseriaceae*; *Neisseria*; *Neisseria elongata* |
| 37 | *Neisseria gonorrhoeae* | NCTC 8375 | X07714 | 88.17 | Bacteria; Proteobacteria; Betaproteobacteria; Neisseriales; *Neisseriaceae*; *Neisseria* |
| 38 | *Herminiimonas contaminans* | CCUG 53591 | HE610501 | 88.17 | Bacteria; Proteobacteria; Betaproteobacteria; Burkholderiales; *Oxalobacteraceae*; *Herminiimonas* |
| 39 | *Herminiimonas saxobsidens* | NS11 | AM493906 | 88.13 | Bacteria; Proteobacteria; Betaproteobacteria; Burkholderiales; *Oxalobacteraceae*; *Herminiimonas* |
| 40 | *Crenobacter luteus* | YIM 78141 | KF771276 | 88.11 | Bacteria; Proteobacteria; Betaproteobacteria; Neisseriales; *Neisseriaceae*; *Crenobacter* |
| 41 | *Chromobacterium amazonense* | CBMAI310 | KF137653 | 88.11 | Bacteria; Proteobacteria; Betaproteobacteria; Neisseriales; *Neisseriaceae*; *Chromobacterium* |
| 42 | *Formivibrio citricus* | DSM 6150 | Y17602 | 88.11 | Bacteria; Proteobacteria; Betaproteobacteria; Neisseriales; *Neisseriaceae*; *Formivibrio* |
| 43 | *Gulbenkiania indica* | HT27 | DQ415656 | 88.10 | Bacteria; Proteobacteria; Betaproteobacteria; Neisseriales; *Neisseriaceae*; *Gulbenkiania* |
| 44 | *Herminiimonas aquatilis* | CCUG 36956 | AM085762 | 88.07 | Bacteria; Proteobacteria; Betaproteobacteria; Burkholderiales; *Oxalobacteraceae*; *Herminiimonas* |
| 45 | *Actimicrobium antarcticum* | KOPRI 25157 | HQ699437 | 87.99 | Bacteria; Proteobacteria; Betaproteobacteria; Burkholderiales; *Oxalobacteraceae*; *Actimicrobium* |
| 46 | *Sutterella parvirubra* | YIT 11816 | AB300989 | 87.98 | *Bacteria*; *Proteobacteria*; *Betaproteobacteria*; *Burkholderiales*; *Sutterellaceae*; *Sutterella* |
| 47 | *Herminiimonas glaciei* | UMB49 | EU489741 | 87.89 | Bacteria; Proteobacteria; Betaproteobacteria; Burkholderiales; *Oxalobacteraceae*; *Herminiimonas* |
| 48 | *Oxalicibacterium horti* | OD1 | AB469786 | 87.89 | Bacteria; Proteobacteria; Betaproteobacteria; Burkholderiales; *Oxalobacteraceae*; *Oxalicibacterium* |
| 49 | *Massilia consociata* | CCUG 58010 | FN814307 | 87.87 | Bacteria; Proteobacteria; Betaproteobacteria; Burkholderiales; *Oxalobacteraceae*; *Massilia* |
| 50 | *Aquaspirillum putridiconchylium* | IAM 14964 | AB076000 | 87.83 | Bacteria; Proteobacteria; Betaproteobacteria; Neisseriales; *Neisseriaceae*; *Aquaspirillum* |
| 51 | *Cupriavidus necator* | N-1 | CP002878 | 87.79 | Bacteria; Proteobacteria; Betaproteobacteria; Burkholderiales; *Burkholderiaceae*;  *Cupriavidus* |
| 52 | *Chromobacterium violaceum* | ATCC 12472 | AE016825 | 87.76 | Bacteria; Proteobacteria; Betaproteobacteria; Neisseriales; *Neisseriaceae*; *Chromobacterium* |
| 53 | *Herminiimonas fonticola* | S-94 | AY676462 | 87.75 | Bacteria; Proteobacteria; Betaproteobacteria; Burkholderiales; *Oxalobacteraceae*; *Herminiimonas* |
| 54 | *Herminiimonas arsenicoxydans* | ULPAs1 | CU207211 | 87.65 | Bacteria; Proteobacteria; Betaproteobacteria; Burkholderiales; *Oxalobacteraceae*; *Herminiimonas* |
| 55 | *Chitinibacter tainanensis* | BCRC 17254 | AY264287 | 87.62 | Bacteria; Proteobacteria; Betaproteobacteria; Neisseriales; *Neisseriaceae*; *Chitinibacter* |
| 56 | *Chromobacterium pseudoviolaceum* | CCM 2076 | AJ871128 | 87.61 | Bacteria; Proteobacteria; Betaproteobacteria; Neisseriales; *Neisseriaceae*; *Chromobacterium* |
| 57 | *Cupriavidus nantongensis* | X1 | CP014844 | 87.58 | Bacteria; Proteobacteria; Betaproteobacteria; Burkholderiales; *Burkholderiaceae*; *Cupriavidus* |
| 58 | *Herbaspirillum chlorophenolicum* | CPW301 | LFLT01000090 | 87.58 | Bacteria; Proteobacteria; Betaproteobacteria; Burkholderiales; *Oxalobacteraceae*; *Herbaspirillum* |
| 59 | *Cupriavidus pauculus* | LMG 3413 | AF085226 | 87.57 | Bacteria; Proteobacteria; Betaproteobacteria; Burkholderiales; *Burkholderiaceae*; *Cupriavidus* |
| 60 | *Cupriavidus metallidurans* | CH34 | CP000353 | 87.56 | Bacteria; Proteobacteria; Betaproteobacteria; Burkholderiales; *Burkholderiaceae*; *Cupriavidus* |
| 61 | *Chromobacterium vaccinii* | MWU205 | JZJL01000120 | 87.55 | Bacteria; Proteobacteria; Betaproteobacteria; Neisseriales; *Neisseriaceae*; *Chromobacterium* |
| 62 | *Thiobacillus thiophilus* | D24TN | EU685841 | 87.51 | Bacteria; Proteobacteria; Betaproteobacteria; Nitrosomonadales; *Thiobacillacaeae*;  *Thiobacillus* |
| 63 | *Cupriavidus plantarum* | ASC-64 | HQ438086 | 87.51 | Bacteria; Proteobacteria; Betaproteobacteria; Burkholderiales; *Burkholderiaceae*;  *Cupriavidus* |
| 64 | *Herbaspirillum huttiense* subsp. *putei* | IAM 15032 | ANJR01000027 | 87.51 | Bacteria; Proteobacteria; Betaproteobacteria; Burkholderiales; *Oxalobacteraceae*; *Herbaspirillum*; *Herbaspirillum huttiense* |
| 65 | *Cupriavidus taiwanensis* | LMG 19424 | CU633749 | 87.51 | Bacteria; Proteobacteria; Betaproteobacteria; Burkholderiales; *Burkholderiaceae*; *Cupriavidus* |
| 66 | *Polynucleobacter cosmopolitanus* | MWH-MoIso2 | AJ550672 | 87.50 | *Bacteria*; *Proteobacteria*; *Betaproteobacteria*; *Burkholderiales*; *Burkholderiaceae*;  *Polynucleobacter* |
| 67 | *Noviherbaspirillum aurantiacum* | SUEMI08 | HQ830497 | 87.43 | Bacteria; Proteobacteria; Betaproteobacteria; Burkholderiales; *Oxalobacteraceae*; *Noviherbaspirillum* |
| 68 | *Herbaspirillum huttiense* subsp*. huttiense* | ATCC 14670 | AB021366 | 87.43 | Bacteria; Proteobacteria; Betaproteobacteria; *Burkholderiales; Oxalobacteraceae; Herbaspirillum;* *Herbaspirillum huttiense* |
| 69 | *Cupriavidus yeoncheonensis* | DCY86 | KF915797 | 87.43 | Bacteria; Proteobacteria; Betaproteobacteria; Burkholderiales; Burkholderiaceae;  *Cupriavidus* |
| 70 | *Aquitalea pelogenes* | P1297 | KC178611 | 87.41 | Bacteria; Proteobacteria; Betaproteobacteria; Neisseriales; *Neisseriaceae*; *Aquitalea* |
| 71 | *Aquitalea denitrificans* | 5YN1-3 | EU594330 | 87.39 | Bacteria; Proteobacteria; Betaproteobacteria; Neisseriales; *Neisseriaceae*; *Aquitalea* |
| 72 | *Cupriavidus alkaliphilus* | ASC-732 | HQ438078 | 87.37 | Bacteria; Proteobacteria; Betaproteobacteria; Burkholderiales; *Burkholderiaceae*;  *Cupriavidus* |
| 73 | *Paraburkholderia kururiensis* | JCM 10599 | BAMQ01000301 | 87.30 | Bacteria; Proteobacteria; Betaproteobacteria; Burkholderiales; *Burkholderiaceae*; *Paraburkholderia* |
| 74 | *Chromobacterium piscinae* | CCM 3329 | AJ871127 | 87.28 | Bacteria; Proteobacteria; Betaproteobacteria; Neisseriales; *Neisseriaceae*; *Chromobacterium* |
| 75 | *Cupriavidus respiraculi* | AU3313 | AF500583 | 87.23 | Bacteria; Proteobacteria; Betaproteobacteria; Burkholderiales; *Burkholderiaceae*; *Cupriavidus* |
| 76 | *Polynucleobacter duraquae* | MWH-MoK4 | CP007501 | 87.15 | Bacteria; Proteobacteria; Betaproteobacteria; Burkholderiales; *Burkholderiaceae*;  *Polynucleobacter* |
| 77 | *Oxalobacter formigenes* | OxB | U49757 | 87.14 | Bacteria; Proteobacteria; Betaproteobacteria; Burkholderiales; *Oxalobacteraceae*; *Oxalobacter* |
| 78 | *Aquitalea magnusonii* | TRO-001DR8 | DQ018117 | 87.14 | Bacteria; Proteobacteria; Betaproteobacteria; Neisseriales; *Neisseriaceae*; *Aquitalea* |
| 79 | *Undibacterium parvum* | CCUG 49012 | AM397629 | 87.13 | Bacteria; Proteobacteria; Betaproteobacteria; Burkholderiales; *Oxalobacteraceae*; *Undibacterium* |
| 80 | *Herbaspirillum frisingense* | GSF30 | AEEC01001472 | 87.09 | Bacteria; Proteobacteria; Betaproteobacteria; Burkholderiales; *Oxalobacteraceae*; *Herbaspirillum* |
| 81 | *Polynucleobacter sinensis* | MWH-HuW1 | LOJJ01000001 | 87.08 | Bacteria; Proteobacteria; Betaproteobacteria; Burkholderiales; *Burkholderiaceae*; *Polynucleobacter* |
| 82 | *Thiobacillus denitrificans* | DSM 12475 | AQWL01000009 | 86.95 | Bacteria; Proteobacteria; Betaproteobacteria; Nitrosomonadales; *Thiobacillacaeae*;  *Thiobacillus* |
| 83 | *Burkholderia pseudomallei* | ATCC 23343 | CWJA01000021 | 86.95 | Bacteria; Proteobacteria; Betaproteobacteria; Burkholderiales; *Burkholderiaceae*; *Burkholderia* |
| 84 | *Burkholderia mallei* | ATCC 23344 | CP000011 | 86.95 | Bacteria; Proteobacteria; Betaproteobacteria; Burkholderiales; *Burkholderiaceae*; *Burkholderia* |
| 85 | *Polynucleobacter yangtzensis* | MWH-JaK3 | LOJI01000001 | 86.94 | Bacteria; Proteobacteria; Betaproteobacteria; Burkholderiales; *Burkholderiaceae*; *Polynucleobacter* |
| 86 | *Polynucleobacter asymbioticus* | QLW-P1DMWA-1 | CP000655 | 86.94 | Bacteria; Proteobacteria; Betaproteobacteria; Burkholderiales; *Burkholderiaceae*; *Polynucleobacter* |
| 87 | *Massilia alkalitolerans* | YIM 31775 | AY679161 | 86.94 | Bacteria; Proteobacteria; Betaproteobacteria; Burkholderiales; *Oxalobacteraceae*; *Massilia* |
| 88 | *Paraburkholderia rhizoxinica* | HKI 454 | FR687359 | 86.88 | *Bacteria*; *Proteobacteria*; *Betaproteobacteria*; *Burkholderiales*; *Burkholderiaceae*; *Paraburkholderia* |
| 89 | *Undibacterium aquatile* | THG-DN7.3 | KM035973 | 86.54 | Bacteria; Proteobacteria; Betaproteobacteria; Burkholderiales; *Oxalobacteraceae*; *Undibacterium* |

**Table S1.** EzBioCloud similarity-based searches against quality-controlled databases of 16S rRNA sequences and pairwise similarity comparing to 16S rRNA gene sequence of P08^T^.

| **Accession number of protein sequence** | **Annotation** |
| --- | --- |
| WP_102951027.1 | 23S_rRNA_(adenine(2503)-C(2))-methyltransferase_RlmN |
| WP_102950961.1 | 2-C-methyl-D-erythritol_2,4-cyclodiphosphate_synthase |
| WP_102952100.1 | 30S_ribosomal_protein_S10 |
| WP_102952103.1 | 30S_ribosomal_protein_S12 |
| WP_102952120.1 | 30S_ribosomal_protein_S13 |
| WP_102952129.1 | 30S_ribosomal_protein_S14 |
| WP_102951724.1 | 30S_ribosomal_protein_S15 |
| WP_102952142.1 | 30S_ribosomal_protein_S19 |
| WP_102952195.1 | 30S_ribosomal_protein_S2 |
| WP_102950986.1 | 30S_ribosomal_protein_S21 |
| WP_102952118.1 | 30S_ribosomal_protein_S4 |
| WP_102952102.1 | 30S_ribosomal_protein_S7 |
| WP_102951575.1 | 3-isopropylmalate_dehydratase_small_subunit |
| WP_102950364.1 | 5-(carboxyamino)imidazole_ribonucleotide_mutase |
| WP_102950468.1 | 50S_ribosomal_protein_L13 |
| WP_102952132.1 | 50S_ribosomal_protein_L14 |
| WP_102952139.1 | 50S_ribosomal_protein_L16 |
| WP_102950918.1 | 50S_ribosomal_protein_L20 |
| WP_102952141.1 | 50S_ribosomal_protein_L22 |
| WP_102951123.1 | 50S_ribosomal_protein_L27 |
| WP_102950769.1 | 50S_ribosomal_protein_L32 |
| WP_102952180.1 | 50S_ribosomal_protein_L33 |
| WP_102951088.1 | 50S_ribosomal_protein_L34 |
| WP_102952121.1 | 50S_ribosomal_protein_L36 |
| WP_102952130.1 | 50S_ribosomal_protein_L5 |
| WP_102951849.1 | acyl-[acyl-carrier-protein]--UDP-N-acetylglucosamine_O-acyltransferase |
| WP_102951158.1 | acyl_carrier_protein |
| WP_102952375.1 | chaperonin_GroEL |
| WP_102950755.1 | dCTP_deaminase |
| WP_102951714.1 | dUTP_diphosphatase |
| WP_102950137.1 | elongation_factor_4 |
| WP_102950183.1 | F0F1_ATP_synthase_subunit_alpha |
| WP_102951040.1 | fructose-bisphosphate_aldolase_class_II |
| WP_102950866.1 | histidine_triad_nucleotide-binding_protein |
| WP_102952003.1 | peptide_chain_release_factor_1 |
| WP_102950598.1 | ribonuclease_PH |
| WP_102952490.1 | ribose-phosphate_pyrophosphokinase |
| WP_102952281.1 | signal_recognition_particle_protein |
| WP_102951876.1 | SsrA-binding_protein |
| WP_102951508.1 | thymidylate_synthase |
| WP_102952354.1 | transcription_termination_factor_Rho |
| WP_102952037.1 | tRNA_guanosine(34)_transglycosylase_Tgt |
| WP_102950874.1 | threonine--tRNA_ligase |
| WP_102951972.1 | succinate_dehydrogenase_flavoprotein_subunit |
| WP_102952168.1 | type_I_glutamate--ammonia_ligase |
| WP_102950181.1 | F0F1_ATP_synthase_subunit_beta |
| WP_102952101.1 | elongation_factor_G |
| WP_102951788.1 | phosphoribosylformylglycinamidine_cyclo-ligase |
| WP_102950934.1 | thioredoxin-disulfide_reductase |
| WP_102950853.1 | translational_GTPase_TypA |
| WP_102951875.1 | glycine--tRNA_ligase_subunit_alpha |
| WP_102952143.1 | 50S_ribosomal_protein_L2 |
| WP_102951971.1 | succinate_dehydrogenase_iron-sulfur_subunit |
| WP_102952359.1 | 50S_ribosomal_protein_L1 |
| WP_102951243.1 | adenylosuccinate_lyase |
| WP_102952147.1 | tRNA_(N6-isopentenyl_adenosine(37)-C2)-methylthiotransferase_MiaB |
| WP_102951604.1 | phosphopyruvate_hydratase |
| WP_102951144.1 | ATP-dependent_Clp_endopeptidase,_proteolytic_subunit_ClpP |
| WP_102950988.1 | proline--tRNA_ligase |
| WP_102951877.1 | ribosome_recycling_factor |
| WP_102951607.1 | CTP_synthetase |
| WP_102951140.1 | oligoribonuclease |
| WP_102952128.1 | 50S_ribosomal_protein_L6 |
| WP_102951723.1 | polyribonucleotide_nucleotidyltransferase |
| WP_102952126.1 | 30S_ribosomal_protein_S5 |
| WP_102952282.1 | 4-hydroxy-3-methylbut-2-enyl_diphosphate_reductase |
| WP_102952052.1 | 50S_ribosomal_protein_L9 |
| WP_102952360.1 | 50S_ribosomal_protein_L11 |
| WP_102951026.1 | nucleoside-diphosphate_kinase |
| WP_102951247.1 | serine--tRNA_ligase |
| WP_102950932.1 | septum_site-determining_protein_MinD |
| WP_102951661.1 | tryptophan_synthase_subunit_alpha |
| WP_102952000.1 | hypothetical_protein |
| WP_102950773.1 | 3-oxoacyl-ACP_reductase |
| WP_102951989.1 | LPS_export_ABC_transporter_ATP-binding_protein |
| WP_102952054.1 | 30S_ribosomal_protein_S6 |
| WP_102951534.1 | ISC_system_2Fe-2S_type_ferredoxin |
| WP_102950920.1 | phenylalanine--tRNA_ligase_subunit_alpha |
| WP_102951560.1 | iron-sulfur_cluster_assembly_protein_IscA |
| WP_102951124.1 | 50S_ribosomal_protein_L21 |
| WP_102951684.1 | adenylosuccinate_synthase |
| WP_102951242.1 | aspartate--tRNA_ligase |
| WP_102950901.1 | translation_initiation_factor_IF-3 |
| WP_102950234.1 | alanine--tRNA_ligase |
| WP_102952179.1 | 50S_ribosomal_protein_L28 |
| WP_102952053.1 | 30S_ribosomal_protein_S18 |
| WP_102952293.1 | phosphoenolpyruvate_synthase |
| WP_102951498.1 | lysine--tRNA_ligase |
| WP_102952472.1 | 30S_ribosomal_protein_S1 |
| WP_102951127.1 | endonuclease_III |
| WP_102951090.1 | membrane_protein_insertion_efficiency_factor_YidD |
| WP_102951606.1 | 3-deoxy-8-phosphooctulonate_synthase |
| WP_102952119.1 | 30S_ribosomal_protein_S11 |
| WP_102950469.1 | 30S_ribosomal_protein_S9 |
| WP_102951505.1 | methionine_adenosyltransferase |
| WP_102952127.1 | 50S_ribosomal_protein_L18 |
| WP_102950309.1 | tRNA_uridine-5-carboxymethylaminomethyl(34)_synthesis_enzyme_MnmG |
| WP_102950534.1 | UDP-N-acetylmuramate--L-alanine_ligase |
| WP_102950962.1 | ribose_5-phosphate_isomerase_A |
| WP_102951541.1 | metal_ABC_transporter_permease |
| WP_102952283.1 | isoleucine--tRNA_ligase |
| WP_102950226.1 | excinuclease_ABC_subunit_B |
| WP_102950824.1 | transketolase |
| WP_102951739.1 | valine--tRNA_ligase |
| WP_102952428.1 | UDP-N-acetylglucosamine_diphosphorylase/glucosamine-1-phosphate_N-acetyltransferase |
| WP_102952376.1 | co-chaperone_GroES |
| WP_102950111.1 | GMP_synthase_(glutamine-hydrolyzing) |
| WP_102951033.1 | ribosome_biogenesis_GTPase_Der |
| WP_102951070.1 | molecular_chaperone_DnaK |
| WP_102952116.1 | 50S_ribosomal_protein_L17 |
| WP_102952423.1 | U32_family_peptidase |
| WP_102950956.1 | methionine--tRNA_ligase |
| WP_102950772.1 | [acyl-carrier-protein]_S-malonyltransferase |
| WP_102950521.1 | phosphoglycerate_kinase |
| WP_102952473.1 | ribonuclease_HII |
| WP_102950186.1 | F0F1_ATP_synthase_subunit_C |
| WP_102952131.1 | 50S_ribosomal_protein_L24 |
| WP_102950466.1 | DNA_gyrase_subunit_A |
| WP_102951841.1 | DNA-directed_RNA_polymerase_subunit_omega |
| WP_102951159.1 | Holliday_junction_branch_migration_DNA_helicase_RuvB |
| WP_102952357.1 | 50S_ribosomal_protein_L7/L12 |
| WP_102952221.1 | 50S_ribosomal_protein_L19 |
| WP_102951590.1 | NADP-dependent_malic_enzyme |
| WP_102951558.1 | Fe-S_protein_assembly_chaperone_HscA |
| WP_102950187.1 | F0F1_ATP_synthase_subunit_A |
| WP_102950681.1 | NADH-quinone_oxidoreductase_subunit_B |
| WP_102952117.1 | DNA-directed_RNA_polymerase_subunit_alpha |
| WP_102951469.1 | tRNA_(adenosine(37)-N6)-threonylcarbamoyltransferase_complex_transferase_subunit_TsaD |
| WP_102952026.1 | cysteine_synthase_A |
| WP_102951843.1 | endopeptidase_La |
| WP_102951146.1 | ATP-dependent_Clp_protease_ATP-binding_subunit_ClpX |
| WP_102950247.1 | glutamine--tRNA_ligase |
| WP_102950689.1 | NADH-quinone_oxidoreductase_subunit_NuoI |
| WP_102950584.1 | inorganic_diphosphatase |
| WP_102951190.1 | YbaB/EbfC_family_nucleoid-associated_protein |
| WP_102952137.1 | 30S_ribosomal_protein_S17 |
| WP_102952222.1 | tRNA_(guanosine(37)-N1)-methyltransferase_TrmD |
| WP_102951766.1 | GTPase_ObgE |
| WP_102952145.1 | 50S_ribosomal_protein_L4 |
| WP_102950980.1 | recombinase_RecA |
| WP_102950807.1 | cell_division_protein_FtsH |
| WP_102951745.1 | ABC_transporter_ATP-binding_protein |
| WP_102951517.1 | 6,7-dimethyl-8-ribityllumazine_synthase |
| WP_102950683.1 | NADH-quinone_oxidoreductase_subunit_D |
| WP_102951251.1 | molecular_chaperone_DnaJ |
| WP_102951029.1 | 4-hydroxy-3-methylbut-2-en-1-yl_diphosphate_synthase |

**Table S2.** List of orthologous protein clusters concatenated for phylogenomic analysis.

| Name of organisms | INSDC | IMG Taxon ID | Genome size (Mb) | G+C (%) | Total genes | Protein coding genes | Genome assembly level |
| --- | --- | --- | --- | --- | --- | --- | --- |
| *Aquella oligotrophica* P08^T^ | CP024847.1 | 2770939448 | 2.82 | 36.43 | 2625 | 2564 | Complete |
| *Alysiella crassa* DSM 2578^T^ | JQKH00000000.1 | 2571042010 | 2.76 | 45.34 | 2781 | 2706 | Draft |
| *Amantichitinum ursilacus* IGB-41^T^ | LAQT00000000.1 | 2648501275 | 4.93 | 60.09 | 4433 | 4366 | Draft |
| *Bergeriella denitrificans* NBRC 102155^T^ | BCWL00000000.1 | 2731957682 | 2.23 | 55.89 | 2168 | 2106 | Draft |
| *Conchiformibius steedae* DSM 2580^T^ | JHZP00000000.1 | 2556921602 | 2.15 | 50.77 | 2134 | 2061 | Draft |
| *Crenobacter luteus* CN10 | LQQU00000000.1 | 2744054573 | 2.85 | 68.30 | 2826 | 2743 | Draft |
| *Eikenella corrodens* ATCC 23834^T^ | ACEA00000000.1 | * | 2.17 | 55.8 | 2127 | 2075 | Draft |
| *Kingella kingae* ATCC 23330^T^ | AFHS00000000.1 | 651324044 | 1.92 | 46.78 | 2156 | 2102 | Draft |
| *Morococcus cerebrosus* CIP 81.93^T^ | JUFZ00000000.1 | 2634166438 | 2.45 | 51.41 | 2423 | 2356 | Draft |
| *Neisseria gonorrhoeae DSM 9188^T^* | QNRU00000000.1 | 2770939566 | 2.14 | 52.42 | 2210 | 2148 | Draft |
| *Populibacter corticis* 15-3-5^T^ | LQXR00000000.1 | 2724679129 | 2.34 | 47.82 | 2542 | 2483 | Draft |
| *Prolinoborus fasciculus* CIP103579^T^ | ONZB00000000.1 | * | 3.45 | 43 | 3454 | 3357 | Draft |
| *Rivicola pingtungensis* DSM 29661^T^ | QJKI00000000.1 | 2770939562 | 3.71 | 62.64 | 3356 | 3280 | Draft |
| *Simonsiella muelleri* ATCC 29453^T^ | CP019448.1 | 2562617187 | 2.34 | 41.49 | 2340 | 2275 | Complete |
| *Snodgrassella alvi* wkB2^T^ | CP007446.1 | 2585427850 | 2.44 | 42.69 | 2393 | 2303 | Complete |
| *Stenoxybacter acetivorans* DSM 19021^T^ | JQKE00000000.1 | 2571042003 | 2.61 | 45.40 | 2502 | 2450 | Draft |
| *Vitreoscilla stercoraria* DSM 513^T^ | ARNN00000000.1 | 2521172651 | 2.58 | 43.90 | 2610 | 2528 | Draft |

**Table S3.** Genome comparisons between *Aquella oligotrophica* gen. nov. sp. nov. (strain P08^T^) and closely related species in the family *Neisseriaceae*. *For type species genomes without IMG Taxon ID, genome information was retrieved from NCBI PGAP.

| **Bacteria** | **Average amino acid identity (AAI) (%)** |
| --- | --- |
| *Alysiella crassa* DSM 2578^T^ | 43.48 |
| *Amantichitinum ursilacus* IGB-41^T^ | 43.96 |
| *Bergeriella denitrificans* NBRC 102155^T^ | 44.00 |
| *Conchiformibius steedae* DSM 2580 | 44.10 |
| *Crenobacter luteus* CN10 | 46.71 |
| *Eikenella corrodens* ATCC 23834^T^ | 44.30 |
| *Kingella kingae* ATCC 23330^T^ | 44.40 |
| *Morococcus cerebrosus* CIP 81.93^T^ | 44.57 |
| *Neisseria gonorrhoeae* DSM 9188^T^ | 44.29 |
| *Populibacter corticis* 15-3-5^T^ | 43.92 |
| *Prolinoborus fasciculus* CIP103579^T^ | 40.62 |
| *Rivicola pingtungensis* DSM 29661^T^ | 45.49 |
| *Simonsiella muelleri* ATCC 29453^T^ | 43.99 |
| *Snodgrassella alvi* wkB2^T^ | 43.77 |
| *Stenoxybacter acetivorans* DSM 19021^T^ | 44.00 |
| *Vitreoscilla stercoraria* DSM 513^T^ | 44.27 |

**Table S4.** AAI values for pairs of genomes between strain P08^T^ and other phylogenetically related genera in the family *Neisseriaceae*.


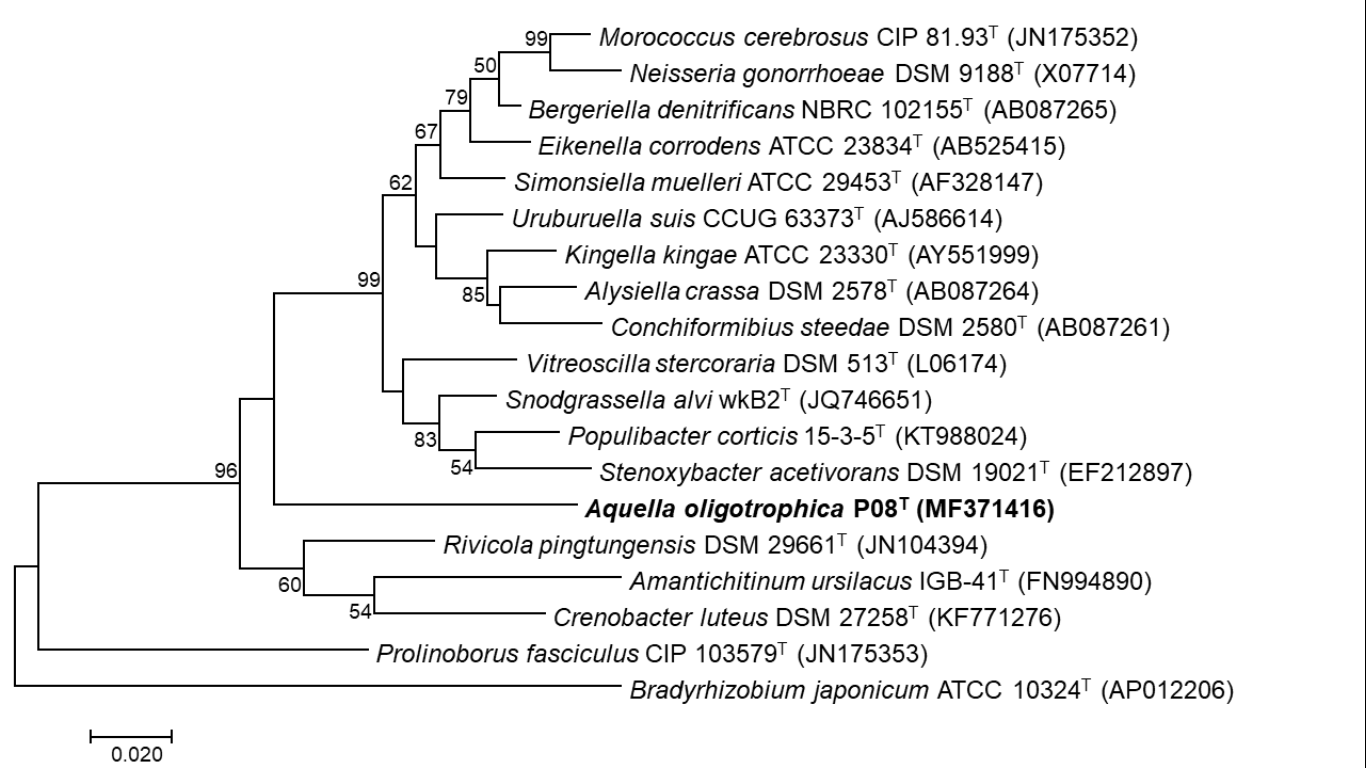


**Figure S1.** Maximum likelihood phylogenetic tree, based on 16S rRNA gene sequences, showing the positions of strain P08^T^ and the most closely related members of the family *Neisseriaceae*. Bootstrap percentage values (1000 replications) greater than or equal to 50 % are shown at nodes. *Bradyrhizobium japonicum* ATCC 10324^T^ (AP012206) was used as an outgroup. Bar, 0.02 substitutions per nucleotide position.


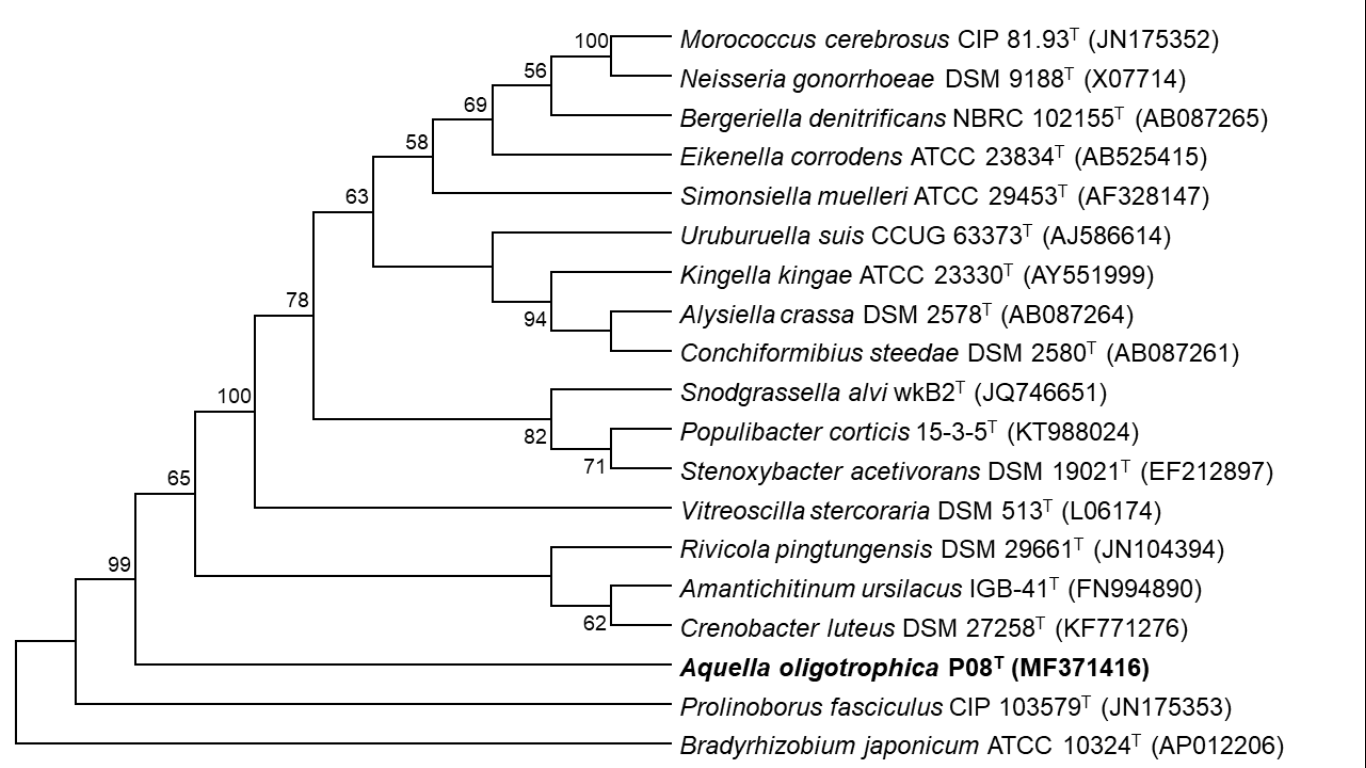


**Figure S2.** Maximum parsimony phylogenetic tree, based on 16S rRNA gene sequences, showing the positions of strain P08^T^ and the most closely related members of the family *Neisseriaceae*. Bootstrap percentage values (100 replications) greater than or equal to 50 % are shown at nodes. *Bradyrhizobium japonicum* ATCC 10324^T^ (AP012206) was used as an outgroup.


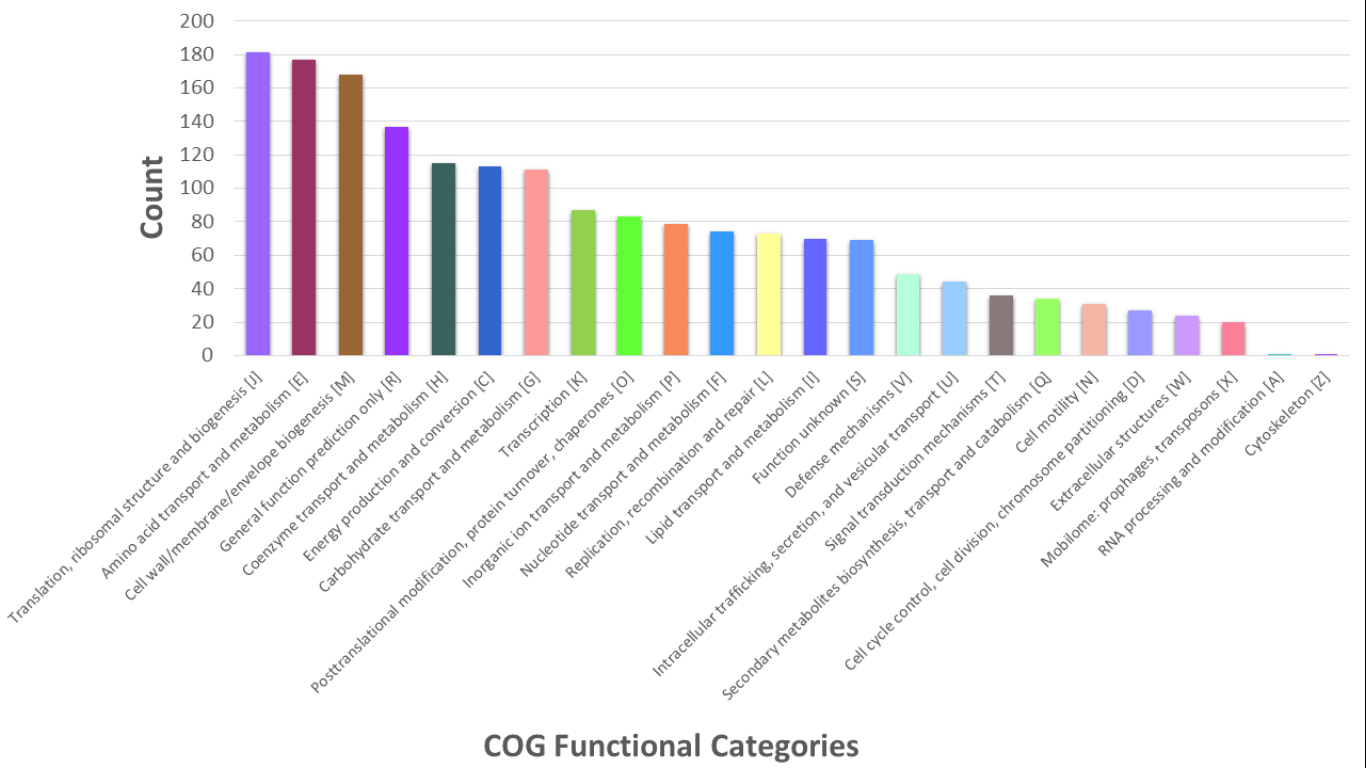


**Figure S3.** Distribution of genes in complete genome of strain P08^T^ into different COGs functional categories.
